# *Notes from the Field*: Knowledge, Attitudes, and Practices Regarding Yellow Fever Vaccination Among Men During an Outbreak — Luanda, Angola, 2016

**DOI:** 10.15585/mmwr.mm6604a6

**Published:** 2017-02-03

**Authors:** Mariel A. Marlow, Maria Augusta Chitula de Feliciano Pambasange, Constantino Francisco, Odete Da Conceiçao Bambi Receado, Maria Jose Soares, Sandra Silva, Carlos Navarro-Colorado, Emily Zielinski-Gutierrez

**Affiliations:** ^1^Epidemic Intelligence Service, CDC; ^2^Division of Foodborne, Waterborne, and Environmental Diseases, National Center for Emerging and Zoonotic Infectious Diseases, CDC; ^3^Angola Field Epidemiology and Laboratory Training Program, Ministry of Health, Republic of Angola; ^4^World Health Organization; ^5^Division of Global Health Protection, Center for Global Health, CDC; ^6^Division of Global HIV and TB – Kenya, Center for Global Health, CDC.

In January 2016, the Angola Ministry of Health reported an outbreak of yellow fever, a vaccine-preventable disease caused by a flavivirus transmitted through the bite of *Aedes* or *Haemagogus* species mosquitoes (*1*,*2*). Although endemic in rural areas of Angola, the last outbreak was in 1988 when 37 cases and 14 deaths were reported (*3*). Large yellow fever outbreaks occur when the virus is introduced by an infected person to an urban area with a high density of mosquitoes and a large, crowded population with little or no immunity (*2*). By May 8, a total of 2,267 suspected cases were reported nationally, of which 696 (31%) were laboratory confirmed; 293 (13%) persons died (*4*). Most (n = 445, 64%) confirmed cases lived in Luanda Province. As part of the public health response that included strengthened surveillance, vector control, case management, and social mobilization (*1*), mass vaccination campaigns were implemented in Luanda during February 2–April 16. Despite >90% administrative vaccination coverage (the number of vaccine doses administered divided by the most recent census estimates for the target population), the province continued to report cases (*4*). Field teams reported low numbers of men being vaccinated, which was a concern because of a preliminary analysis that indicated approximately 70% of confirmed yellow fever cases occurred in males. A rapid assessment to identify and address potential barriers to vaccination among men was designed, using a knowledge, attitudes, and practices survey.

During April 23–25, 2016, a knowledge, attitudes, and practices rapid assessment was administered to men at 10 sites in the four municipalities of Luanda with the greatest number of confirmed cases: Viana, Kilamba Kiaxi, Cacuaco, and Cazenga. The range for administrative vaccination coverage was 22%–137%. Survey sites included public transportation stops, public markets, main streets, and town squares. Interviewers consecutively sampled men of working age while walking in separate trajectories from the site center until the interviewers reached a target of 30 interviews. The questionnaire consisted of multiple choice and open-ended questions on demographics, disease knowledge, vaccination status, vaccination practices, and reasons for nonvaccination, as appropriate.

Overall, 302 men were interviewed. Median age was 30 years (range = 13–68 years); 61% (182) of the men were married or in a domestic partnership. The most frequent occupations reported were street vendor (68, 23%), private business employee (59, 20%), and self-employed (55, 18%). Education levels ranged from illiterate to higher education, with 56% (164) having ≤9 years of formal schooling.

Only 44% of men (133) correctly identified the mosquito as responsible for yellow fever transmission; 15% (48) stated trash/dirty environment, 12% (35) standing/dirty water, and 2% (five) other transmission routes.

Among the 199 (66%) vaccinated men, the majority were vaccinated during the campaign (193, 96%) with events at churches, schools, and neighborhood meetings cited frequently. Among vaccinated men, the most frequently reported sources of information about vaccination were radio (80, 40%), television (78, 39%), and family and friends (64, 32%), with all other sources mentioned by <8%.

When the 103 (34%) unvaccinated men were asked whether they knew where to get vaccinated, 42% (42) answered no. When answering the open-ended question, the most common reasons reported for nonvaccination were lack of time or vaccination conflicting with working hours (26, 25%); thinking the vaccine was dangerous (22, 21%); and not wanting to wait in line (21, 20%) (Figure).

**FIGURE:**
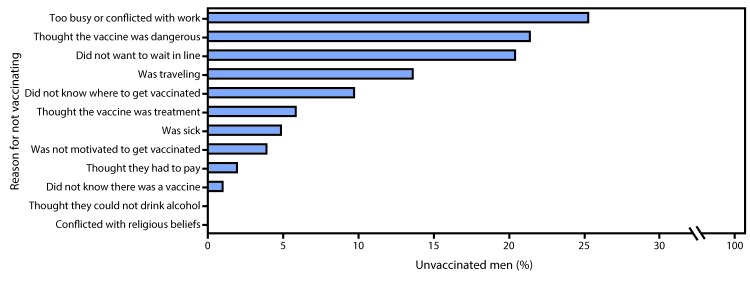
Reasons stated by men for not getting vaccinated during an ongoing outbreak of yellow fever — Luanda, Angola, April 2016

These results highlight several challenges. Most vaccine campaigns target children and women; although this yellow fever campaign needed to reach men, it was not well adapted to their needs. Men could not access vaccination posts during working hours, and those who did experienced long lines because persons from nontargeted municipalities sought vaccination. Lack of information caused many men to fear the vaccine, believing persons had died from the vaccine or that vaccines were fake. Some men did not understand whether the vaccine provided prevention or treatment.

Increased availability of clear information and adaptation of the vaccination activities to the target population’s daily activities were needed. Vaccination campaigns in Luanda were modified to include the following recommendations: diversified modes of communication targeted to men, such as commercials with famous football players; campaigns programmed after working hours and on weekends; door-to-door vaccination in areas with suspected low vaccination coverage; and uniform clear messaging by partners about the critical protection provided by yellow fever vaccination. Messaging also included other ways to prevent infection, such as vector control near dwellings and avoidance of mosquito bites. As of October 20, 2016, no confirmed yellow fever cases have occurred in Angola since June 23, and vaccination campaigns are ongoing in 10 provinces (*5*).

## References

[1] World Health Organization. Emergencies preparedness, response: yellow fever—Angola. Geneva, Switzerland: World Health Organization; 2016. http://www.who.int/csr/don/14-june-2016-yellow-fever-angola/en/

[2] World Health Organization. Yellow fever: fact sheet. Geneva, Switzerland: World Health Organization; 2016. http://www.who.int/mediacentre/factsheets/fs100/en/

[3] World Health Organization. Emergencies: Q&A: yellow fever outbreak in Angola and Democratic Republic of the Congo. Geneva, Switzerland: World Health Organization; 2016. http://www.who.int/emergencies/yellow-fever/mediacentre/qa/en/

[4] World Health Organization. Situation report: yellow fever outbreak in Angola, 8 May 2016. Geneva, Switzerland: World Health Organization; 2016. http://www.afro.who.int/en/yellow-fever/sitreps/item/8620-situation-report-yellow-fever-outbreak-in-angola-8-may-2016.html

[5] World Health Organization. Emergencies: yellow fever situation report. Geneva, Switzerland: World Health Organization; 2016. http://www.who.int/emergencies/yellow-fever/situation-reports/28-october-2016/en/

